# COVID-19-Related Multifocal Demyelinating Neuropathy: Causation or Association

**DOI:** 10.7759/cureus.17770

**Published:** 2021-09-06

**Authors:** Arunmozhimaran Elavarasi, Vasantha Padma Srivastava, Ajay Garg

**Affiliations:** 1 Neurology, All India Institute of Medical Sciences, New Delhi, New Delhi, IND; 2 Neurology, All India Institute, New Delhi, IND; 3 Neuro-radiology, All India Institute of Medical Sciences, New Delhi, New Delhi, IND

**Keywords:** covid-19, madsam, multifocal demyelination, neuropathy, post covid-19 complication

## Abstract

COVID-19 produces pulmonary symptoms in the majority of symptomatic patients. However, demyelinating illnesses of the central and peripheral nervous system are reported in few patients. We report a case of multifocal demyelination involving the peripheral nerves as well as the optic nerve who developed these symptoms while on corticosteroid therapy, which was being given for treating the COVID-19-related inflammatory state. He improved with a short course of steroids. This is the first reported case of multifocal demyelinating neuropathy in relation to COVID-19 to the best of our knowledge. Though there have been reports of COVID-19-associated central nervous system and peripheral nervous system demyelinating illness, whether COVID-19 is causative or just an association is yet to be discerned.

## Introduction

The COVID-19 pandemic has affected more than 119 million people worldwide and caused more than 2.6 million deaths. The disease manifests with features of systemic inflammation such as fever and malaise along with pulmonary symptoms in most symptomatic patients. However, it also can cause an inflammatory syndrome involving multiple systems and can cause neurologic manifestations in a minority [1]. So far, central nervous system (CNS) manifestations such as stroke, ADEM, hemorrhagic leuko-encephalopathy and hypoxic encephalopathy have been reported. In the peripheral nervous system (PNS), Guillain-Barre syndrome (GBS) like presentation seems to be the commonest, though it is not clear if it is causative or just an association [2]. We report a case of multiple mononeuropathies with a demyelinating pattern on electrophysiology following COVID-19 infection in a previously asymptomatic patient who also had optic nerve involvement. To the best of our knowledge, there are no reported cases of Multifocal demyelinating neuropathy associated with COVID-19.

## Case presentation

We report a 53-year-old man who was previously well, developed fever and malaise and on evaluation was found to be positive for SARS-CoV-2 by RT-PCR. He was hospitalized for isolation and observation. His hemogram and metabolic parameters were within normal limits. However, he had systemic inflammation as evidenced by elevated C-reactive peptide (CRP) levels to the tune of 275 mg/L. He was treated with supportive care and prednisolone (30 mg per day) for a week. He had no infiltrates on the chest x-ray. He became afebrile in a few days and over the next two weeks, his CRP levels reached the normal range. On the 17th day after the onset of symptoms, he developed left foot drop; paresthesias over the dorsum of the left foot and the ulnar border of the left upper limb. He reported blurring of vision in the right eye and his acuity was reduced to 6/24 in the right eye. He was not able to dorsiflex his left ankle (MRC 1/5). The rest of the muscle groups had normal power. His deep tendon reflexes were absent except for normal knee and triceps jerks. Postural tremors were observed in the outstretched hands, suggestive of neurogenic tremors seen in demyelinating neuropathy.

He underwent nerve conduction studies which revealed prolonged latencies and conduction block in the left peroneal nerve with reduced SNAPs in the left ulnar nerve (Table 1).

**Table 1:** Nerve conduction studies at onset and at follow up Motor NCS: There was a conduction block with reduced conduction velocity in the left peroneal nerve at the onset. The subsequent NCS done in November 2020 showed nonrecordable potentials in the left peroneal nerve, reduced velocities of both tibial and right peroneal nerves. These abnormalities have significantly improved in the follow-up NCS. The left peroneal CMAPs which were nonrecordable have now become recordable, albeit low. Similarly, sensory nerve latencies have been reduced and velocities normalized on follow-up study.

Motor NCS	October 2020			November 2020			February 2021		
	Onset latency ms	CMAP mV	Conduction velocity m/s	Onset latency ms	CMAP mV	Conduction velocity m/s	Onset latency ms	CMAP mV	Conduction velocity m/s
Left median wrist	Not done	Not done	Not done	3.8	7.8	43	3.65	8.9	52
Left median elbow	Not done	Not done	Not done	9.1	6.4		8.3	7.8	
Right median wrist	Not done	Not done	Not done	3.7	8.8	41	3.02	8.8	52
Right median elbow	Not done	Not done	Not done	9.3	8.7		7.9	8.6	
Left ulnar wrist	Not done	Not done	Not done	2.92	5.8	47	2.08	8.1	51
Left ulnar elbow	Not done	Not done	Not done	8.3	5.5		7.4	7.1	
Right ulnar wrist	Not done	Not done	Not done	2.81	5.8	47	2.34	7	48
Right ulnar elbow	Not done	Not done	Not done	8.85	5.5		7.7	7	
Left tibial ankle	4.9	12.3	40	3.85	5.6	30	4.11	8.5	40
L tibial popliteal fossa	14.8	9.2		17.4	4.4		14.4	5.4	
Right tibial ankle	4.8	16.7	40	6.51	10.6	36	4.79	6.3	41
R tibial popliteal fossa	14.9	9.6		17.7	7		15.1	6.2	
Left peroneal ankle	4.8	2.1	28	NR	NR	NR	4.74	0.7	40
L peroneal fibular head	17.5	0.9		NR	NR	NR	13.3	0.6	
R peroneal ankle	4.4	7.2	41	4.58	3	32	3.54	3.3	41
R peroneal fibular head	13.2	5.8		15.6	2.4		11.9	2.5	
Sensory NCS	October 2020			November 2020			February 2021		
	Peak latency ms	SNAP µV	Conduction velocity m/s	Peak latency ms	SNAP µV	Conduction velocity m/s	Peak latency ms	SNAP µV	Conduction velocity m/s
Left median	Not done	Not done	Not done	4.2	6.9	46	3.4	5.8	56
Right median	Not done	Not done	Not done	3.5	5.4	48	3.33	9.9	56
Left ulnar	Not done	Not done	Not done	3.44	4.6	46	2.81	9.3	56
Right ulnar	Not done	Not done	Not done	3.39	8.4	46	3.02	6	52
Left peroneal	NR	NR	NR	NR	NR	NR	3.13	6.8	61
R peroneal	3.9	6	42	NR	NR	NR	3.39	6.1	62

These features were suggestive of multifocal demyelination. The drop in compound muscle action potential along with conduction block is suggestive of demyelination and not axonal loss. Electromyography was not performed as the patient had a conduction block suggesting a demyelinating pattern. Mononeuritis multiplex can also present with multifocal neuropathy, but it is characterized by an axonal pattern on nerve conduction studies.

Further testing for etiology of demyelinating neuropathy was done with IgG and IgM anti-ganglioside antibody profile which was negative. Serum protein electrophoresis and immune fixation electrophoresis did not reveal any monoclonal band. Quantitative kappa and lambda free light chain assay were normal with a free kappa to lambda ratio of 1.11. Anti-aquaporin-4, Anti-Myelin oligodendrocyte glycoprotein (MOG) antibodies, anti-nuclear antibody (ANA), antibodies against extractable nuclear antigens and anti-nuclear cytoplasmic antibodies (ANCA) were negative, as were hepatitis B surface antigen (HBsAg) and anti-hepatitis C virus (HCV) antibodies. Serum ACE levels were normal and cryoglobulins were negative. MR neurography and MRI brain including optic nerve imaging were done 10 weeks after onset of initial symptoms including which did not reveal any abnormality. Cerebrospinal fluid analysis was not performed.

Treatment

He was treated with a short course of steroids for two weeks (30 mg oral prednisolone for two weeks followed by tapering by 5 mg weekly), which was started two days after the left foot drop, with which he had a near-complete recovery.

Outcome and follow-up

There were no new deficits. Follow up at 10 weeks after the onset of symptoms, he had recovered significantly. His tremors resolved, his left ankle power both plantar and dorsiflexion improved to 4/5; he was able to stand on his heels, however, not able to walk on his heels. The sensory symptoms resolved completely. His visual acuity also reached a baseline of 6/6 on the right and 6/9 on the left eye. His nerve conduction studies also showed improvement (Table 1). At 10-month follow-up, all his symptoms had completely resolved.

## Discussion

COVID-19 is associated with a systemic immune response. Demyelinating neuropathy as in GBS is usually preceded by an upper respiratory or gastrointestinal infection, in most cases of presumed viral etiology. With COVID-19 affecting millions of individuals the world over, it is plausible, that some patients have demyelinating peripheral neuropathy triggered by the infection [3,4]. So far, all the reported cases of COVID-19-related neuropathy have been GBS or Miller Fisher syndrome-like presentation [5]. Multifocal acquired demyelinating sensory and motor neuropathy (MADSAM) is a variant of chronic demyelinating inflammatory neuropathy and is historically known to affect the upper limbs more than the lower limbs, usually insidious in onset and gradually progresses over weeks. In our case, we had an acute onreset presentation with left foot drop, which is atypical for classic MADSAM. There have been previously reported cases of MADSAM involving the lower limb [6]. The disease is treated with steroids and IVIg. In up to 50% of cases, the disease may evolve into a classical CIDP. At 10-month follow-up, symptoms had completely resolved in our patient. Thus this patient had a monophasic event of multifocal demyelination.

Our patient also had visual impairment which recovered with treatment. Clinically, his symptoms were consistent with optic neuritis. Thus, he had multifocal demyelination involving the peripheral nerves as well as the optic nerve, though the patient was being treated with steroids to control the inflammatory state. Though there have been reports of COVID-19 associated CNS and PNS demyelinating illness, whether COVID-19 is causative or just an association is yet to be discerned.

We report this case of multifocal demyelinating neuropathy due to its rarity in association with COVID-19. There are other atypical features as well, including lower limb predominant demyelination as well as associated optic neuritis. It is essential to examine patients for other system involvement in patients with COVID-19 as the syndrome is in many cases overshadowed by the pulmonary findings and other symptoms may go unnoticed. Early diagnosis and treatment of these symptoms would ensure better outcomes.

## Conclusions

COVID-19 is associated with a systemic immune response. MADSAM is a variant of chronic demyelinating inflammatory neuropathy, which affects the upper limbs more than the lower limbs, usually insidious in onset and gradually progresses over weeks. Multifocal demyelination involving the peripheral nerves as well as the optic nerve can occur in COVID-19 even while on treatment with steroids to control the inflammatory state and is usually monophasic as in our case. Though there have been reports of COVID-19-associated with CNS and PNS demyelinating illness, whether COVID-19 is causative or just an association is yet to be discerned.
